# Supplementary material for: Learning curve and surgical outcome of robotic assisted colorectal surgery with ERAS program
Source: Sci Rep. 2022 Nov 29;12:20566. doi: 10.1038/s41598-022-24665-w (PMC9709162; doi:10.1038/s41598-022-24665-w)
Supplement: Supplementary file 2 — Supplementary Information 2. [file 41598_2022_24665_MOESM2_ESM.zip › RAL Raw data-Ñ[▒K/118/HS_ñw╝╨░O▒Kñσ.pdf]

正式摘要(電子簽章：000000227663526)		
W52 / W52 ☐英文標題(注意:無英文姓名-請補病人基本檔!)	☐【讀取】憑證	

臺中榮民總醫院
出院病歷摘要

轉入號 : 轉出號 :

[redacted]

(8)流水編號	
(9)入院日期	110/10/12 (2021) 直腸外科 W52 - 091
(10)轉科日期	
(11)出院日期	110/10/20 (2021) 住院天數計 8 日

診斷

- 入院 : 1.rectal cancer
  2.cervical cancer, Figo stage IB, s/p radical hysterectomy and bilateral salpingo-oophorectomy on 1997 and radiotherapy
- 出院 : 1.rectal cancer s/p robotic LAR+ loop ileostomy on 2021/10/14
  2.cervical cancer, Figo stage IB, s/p radical hysterectomy and bilateral salpingo-oophorectomy on 1997 and radiotherapy

主訴

- diarrhea for about 3 months

病史

- This 69 years old female patient has past history of cervical cancer, Figo stage IB, s/p radical hysterectomy and bilateral salpingo-oophorectomy on 1997 and radiotherapy. She recieved regular pap smear follow-up. She complained defecation change with tenesmus and sometimes diarrhea for about 3 months. She came to LMD for help. Rectal tumor was found in colonoscopy that malignancy was proved in biopsy. She came to our hospital for help. Operation was suggested. Thus, she was admitted for treatment.

體檢發現

-
  - General appearance :
    - easy looking , conscious clear , without cardiopulmonary distress
    - Vital signs自動帶入date 體溫(BT) 脈搏(P) 呼吸(R) 血壓(BP) 身高(BH) 體重(BW) BMI
      20211012 36.6℃ 100次/分 17次/分 129/80mmHg 154.5cm 50.2kg 21.0
  - HEENT :
    - Eyes: no anemic conjunctiva, no icteric sclera, normal EOM
    - ENT: no otorrhea, no rhinorrhea, no erythematous mucosa, no injected tonsil, no deformity
  - Neck: supple, freely movable, trachea midline,no deformity, no engorged jugular vein , no palpable thyroid or lymph node enlargement
  - Chest:
    - Inspection: Grossly normal, symmetric and fully expansion, no accessory muscle used, no operation scar, no spider angiomata, no gynecomastia
    - Palpation: no palpable mass or nodule, no subcutaneous emphysema, no tenderness
    - Ausculation: clear breathing sound
  - Heart:
    - Percussion: normal heart size
    - Ausculation: regular heart beat, normal S1, S2, no gallop, no murmur
  - Abdomen:
    - Inspection: in
      - flat contour, symetrically, no scar, no stria, no dilated veins, no rashes
    - Ausculation: normoactive bowel sound, no abdominal bruits
    - palpation: soft, no muscle guarding or rigidity, no local tenderness, no

- rebounding pain, no palpable mass, no palpable pulsation
    - Percussion: no shifting dulllness, no hepatomegaly, no splenomagaly
-

Peripheral pulsation :	CA	BA	RA	FA	PA	PTA	DPA
Right side :	++	++	++	++	++	++	++/+++
Left side :	++	++	++	++	++	++	++/+++

- Genito-anus: no tenderness or palpable mass lesion or rectal shelf, normal external genitalia, normal anal tone, brown stool

CRS CONDITION

- CEA: 18.84 ng/ml
-
    - Lung

手術日期及方法

- 手術日期及方法20211013 1. Robotic (XI) assisted low anterior resection
  2. Intraoperative colonoscopy : (-).
  3. High ligation (+) : At IMA root. Left colic artery and IMV were also ligated
  4. Take down from the splenic flexure : (+)
  5. Robotic endoGIA (45-4.1) X2 + EEA (29mm) were used
  6. Proximal cut end: 10 cm, distal cut end: 10 cm
  7. Fr. 12 penrose was inserted from LLQ and into pelvic cavity
  8. ADHESION BARRIER FILM was placed below the fascia of the central umbilicus wound

住院治療經過

- After admission, operation with robotic LAR+loop ileostomy was done on 10/14. We let her try diet under ERAS protocol. PRBC 2U was transfused due to anemia(Hb:8.0). Ostomy rod and drainge tube were removed on POD5. Under the relatively stable condition, she was discharged on 10/19.

合併症

- nil

一般檢查

- 累積報告 :

DATE	TIME	ALB	TP	BIL.T	BIL.D	ALKP	AST	ALT	LDH	AMY	LIPASE	NH3	Ferritin	UA
1101013	1638	2.7		0.42			14	8						
1101015	2013	3.3	5.1	0.52	0.17	50	9	5	134					
1101017	2229			0.82				12						

DATE	TIME	WBC	RBC	HGB	HCT	MCV	PLT	BLST	NEUT	BAND	SEG	LYM	MON	EOS	BAS
1101013	1636	5460		9.5	30.5		191				92.0	6.0	1.0	1.0	0.0
1101015	2013	9670	3.70	8.0	25.6	69.2	180		89.9			6.7	1.9	1.4	0.1
1101016	0907			9.8	31.2										
1101017	2229	7900		11.1	34.2		266		84.1			9.1	4.3	2.4	0.1

DATE	TIME	NA	K	CL	BUN	CREAT	eGFR	CA	Mg	P	CRP	ESR	PCT	D-Dimer	LACTATE
1101013	1637														15.0
1101013	1638	140	2.8		14	0.72	85.36								
1101015	2013	139	3.5	106	16	0.63		8.4							
1101017	2229	137	4.2		11	0.59	107.42								

DATE	TIME	SP-GR	PH	PROT	GLUC	KETON	BILI	UROB	OB	NITR	LEU	RBC	WBC	EPCEL
1101017	2229	1.014	6.5	1+	Negative	Trace	Negative	0.2	3+	Negative	3+	+	+	0-1

DATE	TIME	PH	BEB	SCVO2	PO2	HCO3	HGB	PCO2	TCO2
1101013	1636	7.406	-0.2	99.7	147.8	24.5		39.9	25.7

特殊檢查

病理報告

其它

出院時情況

- 3.改門診治療

出院指示

藥名	劑量	劑量單位	用法	頻次	數量	數量單位	現況	天數	單位
$Biofermin tab	2	TAB	PO	TID	42	TAB	TH	7	天
Acetal tab 500mg	1	TAB	PO	Q6HPRN	28	TAB	TH	7	天
Dobecon tab 100mg	1	TAB	PO	TIDAC	21	TAB	TH	7	天
(□)Takepron OD tab 30mg	1	TAB	PO	QDAC	7	TAB	TH	7	天
120mL (C) B.M. sol	5	ML	PO	TID	1	ML	TH	7	天

出院計劃

主治醫師_2062F 蔣鋒帆 / 住院醫師_2331F 陳凱祥

正式摘要(電子簽章：000000227663526)

W52 / W52

doctorRank:B
